# Tribological Performance and Enhancing Mechanism of 3D Printed PEEK Coated with In Situ ZIF-8 Nanomaterial

**DOI:** 10.3390/polym16081150

**Published:** 2024-04-19

**Authors:** Xinchao Wang, Jiale Hu, Jiajia Liu, Yixin Liang, Lan Wu, Tie Geng, Shihua Liu, Yonggang Guo

**Affiliations:** School of Mechanical & Electrical Engineering, and Henan Provincial Engineering Research Centre of Automotive Composite Materials, Henan University of Technology, Zhengzhou 450001, China; xinchaowang2016@haut.edu.cn (X.W.); jialehu@stu.haut.edu.cn (J.H.); liujiajia@stu.haut.edu.cn (J.L.); 2023930670@stu.haut.edu.cn (Y.L.); fangchenyang@stu.haut.edu.cn (T.G.); liugd2020@haut.edu.cn (S.L.); gyg@haut.edu.cn (Y.G.)

**Keywords:** 3D printing, PEEK, ZIF-8, surface modification, tribological properties

## Abstract

Polyether ether ketone (PEEK) is esteemed as a high-performance engineering polymer renowned for its exceptional mechanical properties and thermal stability. Nonetheless, the majority of polymer-based lubricating materials fail to meet the contemporary industrial demands for motion components regarding high speed, heavy loading, temperature resistance, and precise control. Utilizing 3D printing technology to design and fabricate intricately structured components, developing high-performance polymer self-lubricating materials becomes imperative to fulfill the stringent operational requirements of motion mechanisms. This study introduces a novel approach employing 3D printing technology to produce PEEK with varying filling densities and conducting in situ synthesis of zeolitic imidazolate framework (ZIF-8) nanomaterials on its surface to enhance PEEK’s frictional performance. The research discusses the synthetic methodology, characterization techniques, and tribological performance evaluation of in situ synthesized ZIF-8 nanomaterials on PEEK surfaces. The findings demonstrate a significant enhancement in frictional performance of the composite material under low-load conditions, achieving a minimum wear rate of 4.68 × 10^−6^ mm^3^/N·m compared to the non-grafted PEEK material’s wear rate of 1.091 × 10^−5^ mm^3^/N·m, an approximately 1.3 times improvement. Detailed characterization and analysis of the worn surface of the steel ring unveil the lubrication mechanism of the ZIF-8 nanoparticles, thereby presenting new prospects for the diversified applications of PEEK.

## 1. Introduction

In recent years, the issue of energy and resources has become increasingly crucial due to the rapid growth of the global population, resulting in a steady annual increase in energy demand, estimated at approximately 400 exajoules [1,2]. Consequently, the development of polymers with exceptional friction and wear performance has become indispensable. Polyether ether ketone (PEEK) is a thermoplastic polymer known for its high performance, and it has been widely recognized and utilized in various engineering sectors [3]. Its outstanding mechanical properties, including high strength, excellent chemical resistance, and exceptional thermal stability, make it a sought-after material in industries such as aerospace, automotive, electronics, and medical devices. However, despite its remarkable characteristics, PEEK often faces limitations in terms of friction and wear properties, which can hinder its extensive utilization.

In engineering applications, friction and wear play a crucial role in determining material performance and durability. For PEEK, particularly in situations involving sliding or rolling contact, its inherent limitations in tribological properties become apparent. The increase in friction and wear rates can lead to adverse consequences such as energy losses, component failure, and reduced material lifespan. Therefore, there is a growing need to enhance the tribological properties of PEEK to meet the demands of modern engineering challenges.

Over the years, researchers have explored various methods to improve the friction and wear resistance of polymers. Surface modification of engineering plastics is one of the methods to improve the wear resistance of engineering plastics. Wakelin et al. [4] and Powles et al. [5] employed ion implantation modification techniques, using N^+^ and H^+^ ions, respectively, to modify the surface of PEEK. The research findings indicate that the modified surface of PEEK becomes harder, denser, and exhibits greater elastic recovery. Khare et al. utilized radiation cross-linking modification techniques to explore the effects of γ radiation on the thermal, physical, and frictional characteristics of PEEK, and their study revealed improved frictional performance of PEEK after irradiation [6]. Zhang et al. utilized low-temperature plasma modification techniques with Ar, N_2,_ and O_2_ plasma gases to activate the PEEK surface. The results showed that the plasma treatment enhanced the bonding strength of PEEK [7]. Su utilized physical–chemical deposition modification methods to enable the in situ formation of an a-C:H film with a transitional layer directly on the PEEK surface [8]. This exhibited favorable mechanical properties, adhesive strength, interface stability, frictional wear mechanisms, and wear-resistant protective characteristics. In this study, chemical deposition technology was used to grow a nanomaterial on the surface of PEEK. In situ synthesis (a technique for directly growing nanomaterials on a substrate surface) has gained popularity as an effective method for modifying materials. Among the diverse range of nanomaterials, zeolitic imidazolate frameworks (ZIFs) have garnered attention as promising options for tribological uses. These frameworks, a subset of metal–organic frameworks (MOFs), create porous three-dimensional structures by coordinating metal ions with organic linkers. Among the ZIFs, zeolitic imidazolate framework-8 (ZIF-8) is particularly notable for its exceptional stability, adjustable pore size, and straightforward synthesis process [9,10]. By directly growing ZIF-8 nanomaterials on the surface of PEEK, the lubrication effect of nanomaterials reduces frictional contact and minimizes wear, thereby enhancing the overall frictional performance of PEEK-based components.

This paper explores the method of in situ synthesis of ZIF-8 nanomaterials on the surface of PEEK to enhance its tribological performance. By altering the surface properties of PEEK with nanomaterials, composite materials with superior tribological characteristics can be fabricated. The study primarily investigates the in situ synthesis of zeolitic imidazolate framework-8 (ZIF-8) nanomaterials on the surface of PEEK, analyzes the morphological and structural features of modified PEEK, and investigates its frictional behavior. The successful introduction of ZIF-8 nanomaterials is expected to provide significant avenues for improving the friction and wear properties of PEEK-based materials in a variety of engineering contexts.

## 2. Materials and Methods

### 2.1. Materials and Equipment

Polyether ether ketone (PEEK, PF series-550 PF) was provided by Jilin Zhongyan High Polymer Material Co., Ltd. (Jilin, China). Sulfuric acid (H_2_SO_4_, 98%) was acquired from Chuandong Chemical Co., Ltd., based in Shanghai, China. Methanol, zinc nitrate hexahydrate (Zn(NO_3_)_2_·6H_2_O), and 2-methylimidazole (Hmim) were sourced from Aladdin Industrial Corporation in Shanghai, China. Friction Tester (Jinan Yihua Science and Technology Co., Ltd., Jinan, China), Twin-Screw Extruder (Wuhan Ruiming Testing Instrument Manufacturing Co., Ltd., Wuhan, China), and a 3D printer (MAGIC-HT-L) were purchased from Dongguan Yimai Intelligent Technology Co., Ltd., Dongguan, China.

### 2.2. Preparation of PEEK Samples

Initially, PEEK powder underwent drying in an oven at 120 °C for a duration of 10 h. Subsequently, the dried powder was extruded into filaments, and PEEK samples were fabricated using a MAGIC-HT-L 3D printer employing fused deposition modeling (FDM) technology. Filaments for FDM were manufactured using a twin-screw extruder, with temperature settings of 320 °C, 380 °C, 360 °C, and 355 °C for zones one, two, three, and the nozzle, respectively. The extruded filament had a diameter of approximately 1.75 mm.

The parameters of 3D printing are shown in Table 1. Rectangular blocks with dimensions of 65 mm × 13 mm × 4.2 mm were printed and then each cut into the size of 10 mm × 19 mm × 4 mm. These samples were subsequently polished using 1000 mesh metallographic sandpaper.

### 2.3. Ultrasonic-Assisted Synthesis of PEEK@ZIF-8

**Figure 1:**
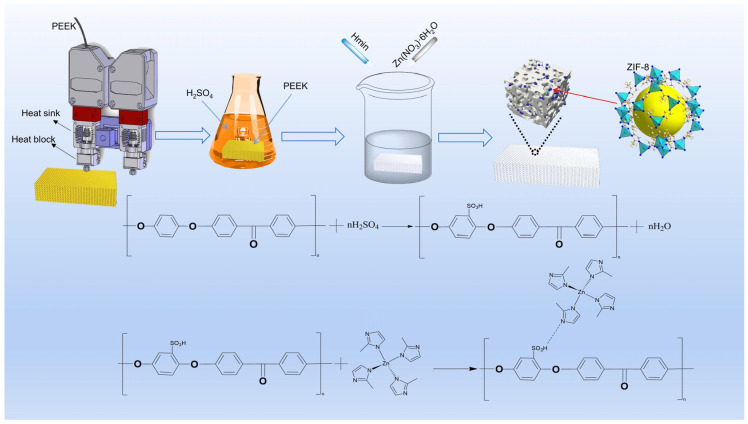
Schematic diagram of the procedure for fabricating SPZ friction test samples.

Figure 1 illustrates the fabrication process and reaction principle of the SPZ friction test samples. First, the 10 mm × 19 mm × 4 mm PEEK samples, fabricated through 3D printing, underwent ultrasonic cleaning with isopropanol, followed by drying in a vacuum oven at 80 °C for 4 h. The dried PEEK samples were then sulfonated by placing them in concentrated sulfuric acid under magnetic stirring, resulting in a uniform porous structure. The sulfonated PEEK (SP) underwent multiple wash cycles (each lasting 10 min) using deionized water and absolute ethanol until reaching a pH of approximately 7. Subsequently, it was dried in a 60 °C oven for a period of 12 h.

SP@ZIF-8 was synthesized using an in situ growth approach. Separately, 2-methylimidazole (0.4915 g) and Zn(NO_3_)_2_·6H_2_O (0.891 g) were dissolved in 50 mL of methanol. The PEEK specimen was placed vertically in a beaker filled with the zinc salt solution. Subsequently, a 2-methylimidazole solution was added to the mixture under ultrasonic agitation for 25 min. The resulting mixture was then allowed to stand overnight in a water bath at 30 °C. Following that, the resulting SP@ZIF-8 (SPZ) underwent three rinses with methanol and was then dried at 60 °C to prepare for subsequent applications.

### 2.4. Characterization

Scanning electron microscopy (SEM, JSM-7900F, Tokyo, Japan) was employed to examine the morphology of worn surface and counter steel. Prior to SEM observation, a thin layer of platinum was sputter-coated onto the sample surfaces to enhance conductivity. X-ray diffraction (XRD) analysis was conducted using a Bruker D8 Advance instrument from Germany to examine the crystalline structure of samples across a 2θ range, spanning 5 °C to 50 °C. Fourier-transform infrared spectroscopy (FTIR, NEXUS 670 spectrometer, Berlin, Germany) was employed for identifying functional groups in the ZIF-8, PEEK, SP, and SPZ samples. The thermogravimetric analyzer (TGA) (STA449F3, Bavaria, Germany) was used to analyze the samples under a nitrogen (N_2_) atmosphere. The samples were placed in alumina crucibles and heated at a rate of 10 K/min. X-ray photoelectron spectroscopy (XPS) using the Axis Supra instrument from the UK was employed to determine the chemical composition of the worn surface.

**Figure 2:**
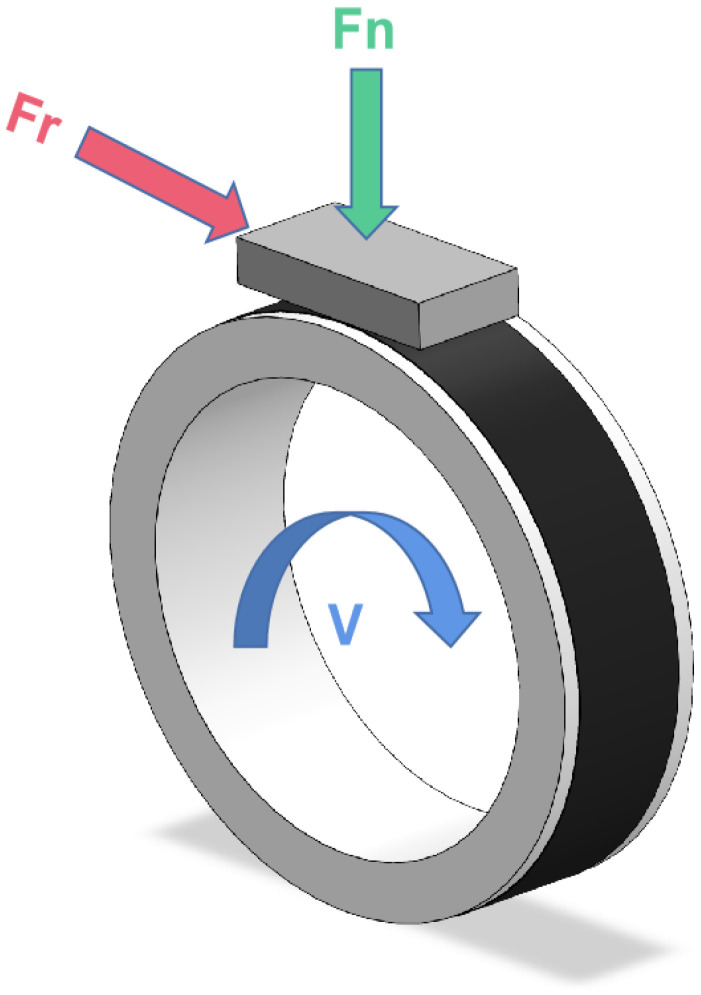
The Block-on-Ring sliding contact configuration.

The tribological properties of PEEK composite materials were tested on a BMDW-1A multi-condition (Block-on-Ring) friction and wear tester (Jinan Boyuan Huachuang Intelligent Technology Co., Ltd., Jinan, China). The schematic diagram of the contact configuration is shown in Figure 2. The dimensions of the block-shaped polymer samples were as described in Section 2.2. Ring-shaped bearing steel (GCr15) was used as the counterpart material [11]. Prior to each test, the GCr15 mating ring underwent grinding with 1000 mesh sandpaper to attain an average surface roughness (Ra) ranging between 0.2 and 0.3 μm. Prior to each test, both the mating ring and the polymer specimen were thoroughly cleaned in isopropanol using ultrasonic agitation for 10 min. Friction and wear tests lasted for 2 h in dry sliding conditions at room temperature. During the friction process, the friction coefficient (COF) and specific wear rate (Ws) are pivotal parameters reflecting the tribological performance of a material. The COF is calculated using sensors and statistical software (MMW2, 1.00) attached to a high-speed block-on-ring friction testing machine. The Ws, which represents the volume of wear per unit load and unit sliding distance for polymer materials, is determined by measuring the length and width of wear tracks, and then calculating the material’s specific wear rate using Formula (1). Each set of parallel experiments is repeated at least three times, and the average values of COF and Ws are taken.

(1) $$Ws=L^{\prime }\times \left[\left.R^{2}arcsin\left(\frac{W}{2R}\right.\right)-\frac{W}{4}\left.\sqrt{{4R}^{2}-W^{2}}\right.\right]/FL\left({\mathrm{mm}}^{3}/N\cdot m\right)$$

where L′ is the width of the specimen (mm), R is the diameter of the counter steel ring (mm), W is the width of the wear track (mm), F is the applied normal force (N), and L is the sliding distance (m).

## 3. Results and Discussion

### 3.1. Microstructural Characterization of PEEK Surface Modification

**Figure 3:**
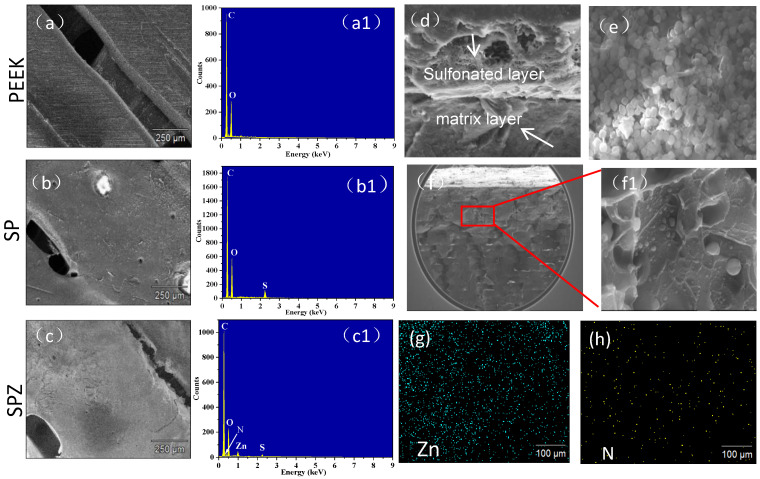
SEM images of PEEK (**a**), SP (**b**), SPZ (**c**–**e**), and the corresponding line scan EDS spectra for PEEK (**a1**), SP (**b1**), and SPEEK@ZIF-8 (**c1**), as well as the cross-sectional image of SPZ (**f**,**f1**) and the surface scanning EDS spectrum of SPZ (**g**,**h**).

Figure 3 illustrates the morphological changes on the surface of PEEK before and after in situ growth of the nanoparticles. It can be seen that the original scratches induced by grinding (Figure 3a) disappeared with surface sulfonation (Figure 3b), which provided active sites for the following in situ growth of nanoparticles [12]. After the in situ reaction (Figure 3), the surface of the sulfonated PEEK was covered with a thin film (Figure 3d), and the thickness of the film was closely related to the sulfonation time. The sulfonation layer exhibits a complex network of pores, clearly demarcated from the PEEK matrix. Elemental analysis revealed that sulfonation and in situ nanoparticle growth altered the elemental composition of the PEEK surface, transforming it from C and O to C, O, S, and C, O, S, N, Zn, respectively (Figure 3(a1–c1)). Surface morphology analysis of the film, as shown in Figure 2, indicates that it was composed of dodecahedral-shaped particles with dimensions of approximately 500 nm (Figure 3e), a morphology similar to typical ZIF-8 particles [13]. From the cross-sectional image of SPZ (Figure 3f), it can be seen that the concentrated sulfuric acid penetrates into the voids of PEEK. From the internal cross-sectional view (f1), it is evident that ZIF-8 particles were generated inside the PEEK matrix, and their shape differences were related to the concentration of Hmim [14]. Elemental surface distribution showed the uniform presence of N and Zn from ZIF-8 (Figure 3g,h). Based on the aforementioned morphology and elemental analysis results, it can be preliminarily inferred that ZIF-8 had successfully grown in situ on the surface of PEEK.

### 3.2. Phase Characterization Analysis

**Figure 4:**
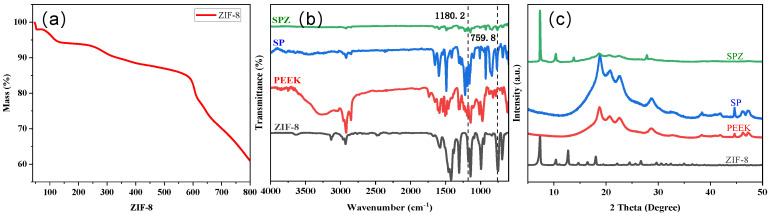
The TGA curve of ZIF-8; ATR−FT−IR spectra and XRD spectra of PEEK, SP, ZIF-8, and SPZ samples.

Figure 4a displays the thermogravimetric analysis (TGA) of ZIF-8 under a nitrogen flow. The weight loss from room temperature to 100 °C can be attributed to the removal of guest molecules and adsorbed gases. Furthermore, a relatively minor weight loss was seen between 100 °C and 600 °C, indicating the high thermal stability of ZIF-8. However, the weight loss rate of ZIF-8 was significantly increased at a temperature beyond 600 °C due to the collapse of its framework and decomposition [15].

Figure 4b displays the ATR-FTIR spectra of ZIF-8, PEEK, SP, and SPZ. Regarding ZIF-8, the peaks at 1180.2 cm^−1^ and 1585 cm^−1^ were attributed to C=N stretching vibrations possibly linked with the 2-methylimidazole organic ligand [16]. Furthermore, the absorption peaks at 1382 cm^−1^ and 1423 cm^−1^ possibly corresponded to stretching vibrations of the imidazole ring [17]. Peaks at 694.3 cm^−1^ and 759.8 cm^−1^ may be associated with SP^2−^C−H bending vibrations, consistent with reported C-H bending modes [16]. Regarding SP, the absorption peaks at 1080 cm^−1^ and 1250 cm^−1^ may be related to the asymmetric stretching vibration of O=S=O and the symmetric stretching vibration of S=O, respectively, indicating the possibly introduction of -SO_3_H groups to the surface during the sulfonation process [18].

To characterize the crystal structures of ZIF-8, PEEK, SP, and SPZ, XRD analysis was conducted, and the corresponding results are displayed in Figure 4c. The XRD pattern of ZIF-8, possessing a (SOD) topology structure, exhibits diffraction peaks at 2θ = 6.92°, 9.68°, and 12.02°, corresponding to the (011), (002), and (112) crystal planes of ZIF-8, respectively, which are in excellent agreement with previously reported results [19]. In the XRD pattern of SPZ, diffraction peaks associated with the crystal planes of ZIF-8 and PEEK were identified. Specifically, the peaks at 2θ = 6.92°, 12.02°, 18.62°, and 22.52° correspond to the characteristic peaks of ZIF-8 crystal and the (110), (220) crystal plane diffractions of PEEK, respectively. Additionally, absorption peaks corresponding to ZIF-8, PEEK, and SP were observed in the ATR spectrum of SPZ, confirming the effective deposition of ZIF-8 on the substrate.

### 3.3. Friction and Wear Properties

#### 3.3.1. Influence of in-Filling Density on Tribological Performance

**Figure 5:**
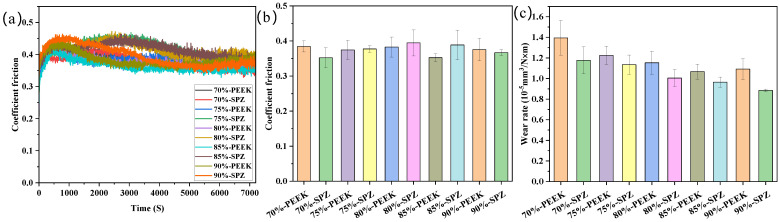
Time-dependent friction coefficient curves (**a**) and steady−state friction coefficient plots (**b**) for PEEK and SPZ at different filler densities, along with the characteristic wear rates (**c**). Load: 80 N, rotational speed: 1 m/s.

Figure 5 provides information on the friction characteristics of the surface-modified material SPZ. Figure 5a describes the variation of friction coefficient over time for different fill densities of PEEK and SPZ. These curves show a trend of initially increasing, then decreasing, and finally stabilizing friction coefficients. Among them, the friction curves of SPZ with fill densities of 75%, 80%, and 85% exhibit a longer running-in period, reaching their peak at approximately 3000 s. In contrast, the friction samples of different fill densities of PEEK reach their peak at 500 s, followed by a decrease in friction coefficient, stabilizing at around 2500 s. From the friction curves, their final stable values are very close, indicating that the friction coefficient of PEEK samples is not significantly affected by the increase in fill density. Among them, the friction coefficient of 85% PEEK is the lowest. The initial rise in friction coefficient can be explained by the gradual increase in the contact area between the steel ring and the sample as friction begins. As the experiment progresses, nanoparticles gradually release from the PEEK matrix to the friction interface, interacting with wear debris from the PEEK matrix, forming a transfer film. This film alters the nature of friction, transitioning it from friction between the steel ring and the sample to friction among the steel ring, transfer film, and composite material. This further leads to the gradual stabilization of the friction coefficient. When examining PEEK and SPZ with different in-filling densities, the steady-state friction coefficient of PEEK does not show significant variation (Figure 5b). However, the steady-state friction coefficient of SPZ at different filler densities exhibits a trend of initially increasing and then decreasing, with the maximum value occurring at an 80% filler density. As illustrated in Figure 5c, an increase in filler density corresponds to a decreasing trend in the wear rates of PEEK and SPZ. Notably, SPZ exhibits a more pronounced reduction compared to PEEK. This phenomenon can be attributed to the incorporation of nanoparticles into the surface interstices of the samples during the friction process, enhancing their load-bearing capacity and consequently mitigating wear rates.

**Figure 6:**
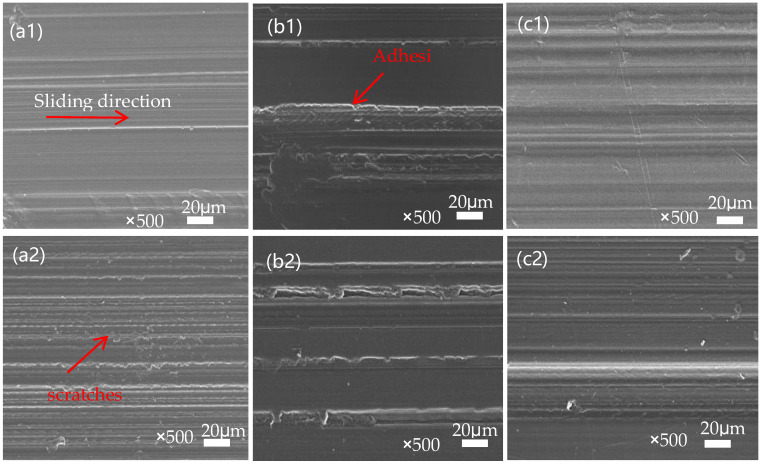
SEM images of the post−wear surfaces of different filler densities of PEEK and SPZ in contact with GCr15. (**a1**) 70% PEEK; (**b1**) 80% PEEK; (**c1**) 90% PEEK; (**a2**) 70% SPZ; (**b2**) 80% SPZ; (**c2**) 90% SPZ.

The morphology of the worn surfaces of different filler densities of PEEK and its SPZ composites under dry friction conditions is shown in Figure 6. From Figure 6(a1–c1), it can be seen that the worn surface of pure PEEK was covered with numerous grooves accompanied by adhesive signs, which corresponded to a relatively higher wear rate [20]. From Figure 6(a2–c2), it can be seen that, after surface grafting, the worn surfaces of the SPZ materials became relatively flat with fewer and shallower furrows, inside which fine scratches induced from the in situ formed ZIF-8 particles were present. A higher filler density implies that the gaps on the sample surface are more easily filled with wear debris and nanoparticles, which enhances their load-bearing capacity and reduces the wear rate. This can be attributed to the good load-bearing capacity of ZIF-8 nanoparticles, which helps reduce frictional wear. Additionally, the nanoparticles, once released into the friction interface, move in a rolling manner thus reducing the actual contact area and promoting the formation of a high-quality transfer film. This also highlighted the role of ZIF-8 particles in modifying the friction and wear behavior of the studied materials.

**Figure 7:**
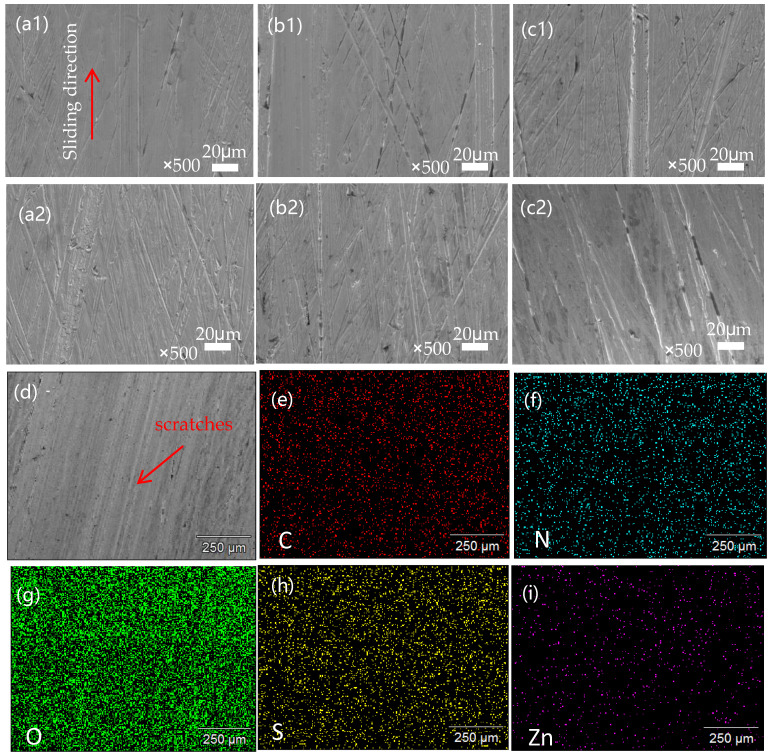
Microscopic morphology and EDS analysis of the transfer films formed on PEEK and SPZ in contact with GCr15 after wear. (**a1**) 70% PEEK; (**b1**) 80% PEEK; (**c1**) 90% PEEK; (**a2**) 70% SPZ; (**b2**) 80% SPZ; (**c2**) 90% SPZ; SPZ (**d**–**i**).

Figure 7 depicts the surface morphology of transfer films formed on the counter steel rings. High-quality transfer films helped reduce the scratching and cutting actions of the steel rings’ surface on the polymer materials, thereby influencing the tribological performance [21]. It is clear that the quality of the transfer films was influenced by the in situ formation of ZIF-8 particles on PEEK’s surface. When slid against PEEK with different in-filling densities, there was hardly any formation of transfer films on the counter ring surface, as can be seen from Figure 7(a1–c1). In contrast, the in situ formed ZIF-8 particles helped the formation of relatively uniform transfer films, with which the cutting and abrasive action of the counter rings was abated. Moreover, with a higher number of ZIF-8 particles in PEEK printed with lower in-filling densities, the counter ring surface appeared to be smoother. From the different examples shown, the transfer film formed between the steel ring and the material surface varies in quality. In some cases (Figure 7(a1–c1)), the transfer film quality is poor and uneven, failing to protect the material matrix effectively, leading to an exacerbation of cutting and scratching effects from the steel ring, and the surface becomes rougher.

Improved conditions are demonstrated after modification (a2–c2 in Figure 7), where the in situ grafting of ZIF-8 nanoparticles on the PEEK surface enhances the quality of the transfer film, and it becomes relatively uniform. This improvement effectively reduces the cutting action of the steel ring on the PEEK matrix. Concurrently, with the escalating filler density, there is a corresponding increase in the quantity of nanoparticles transferred onto the surface of the transfer film. This occurrence is attributed to the higher filler density, which facilitates a more efficient filling of nanoparticles and wear debris, resulting in a smoother and more even surface.

To gain a deeper understanding of the distribution and role of the ZIF-8 nanoparticles in the transfer film, spectral analysis was performed. It can be seen from the spectra shown in Figure 7e–i that there is a significant presence of C, O, N, S, and Zn elements in the transfer film. This indicates that ZIF-8 nanoparticles were successfully grafted onto the PEEK surface, and these particles played a synergistic role in the formation of the transfer film. Specifically, ZIF-8 particles were ground and embedded into the transfer film, thereby improving the tribological performance of the composite material.

#### 3.3.2. Impact of Sulfonation Time on Tribological Performance

**Figure 8:**
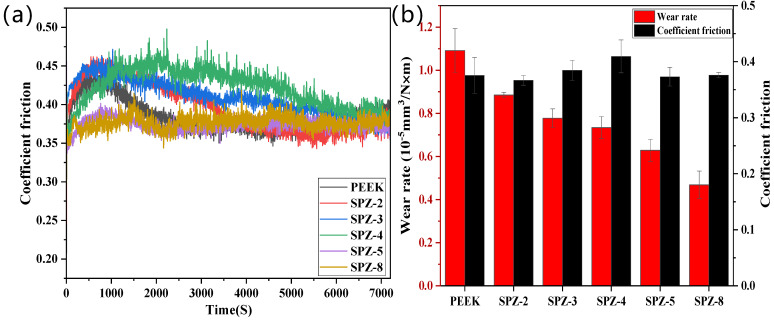
The friction coefficient curves over time (**a**) and steady−state friction coefficient plots with characteristic wear rates (**b**) for PEEK and SPZ with different sulfonation times. Load: 80 N, rotational speed: 1 m/s.

Figure 8 illustrates the impact of surface modification through different sulfonation times on the frictional characteristics of pure PEEK and PEEK with various sulfonation times. From Figure 8a, it can be observed that the friction coefficient curves of SPZ follow the same trend as those of PEEK. However, the running-in period of the friction curves for the sulfonation times of 2 min, 3 min, and 4 min is longer, with the friction curve for sulfonation at 4 min only reaching a steady state at 5500 s. The friction coefficient curves of SPZ for the sulfonation times of 5 min and 8 min are relatively stable compared to curves for other sulfonation times. Figure 8b further revealed that the steady-state friction coefficient of SPZ varied with the sulfonation time, showing an initial increase followed by a decrease and eventual stabilization. Additionally, it is evident that the wear rate of SPZ was significantly decreased with increasing sulfonation time. For example, SPZ with a sulfonation time of 8 min exhibited a wear rate of 4.68 × 10^−6^ mm^3^/N·m, while pure PEEK showed a wear rate of 1.091 × 10^−5^ mm^3^/N·m. This can be attributed to the increased grafting of nanoparticles onto the PEEK’s surface with prolonged sulfonation time, with which the load-carrying capacity of the PEEK’s surface was enhanced.

**Figure 9:**
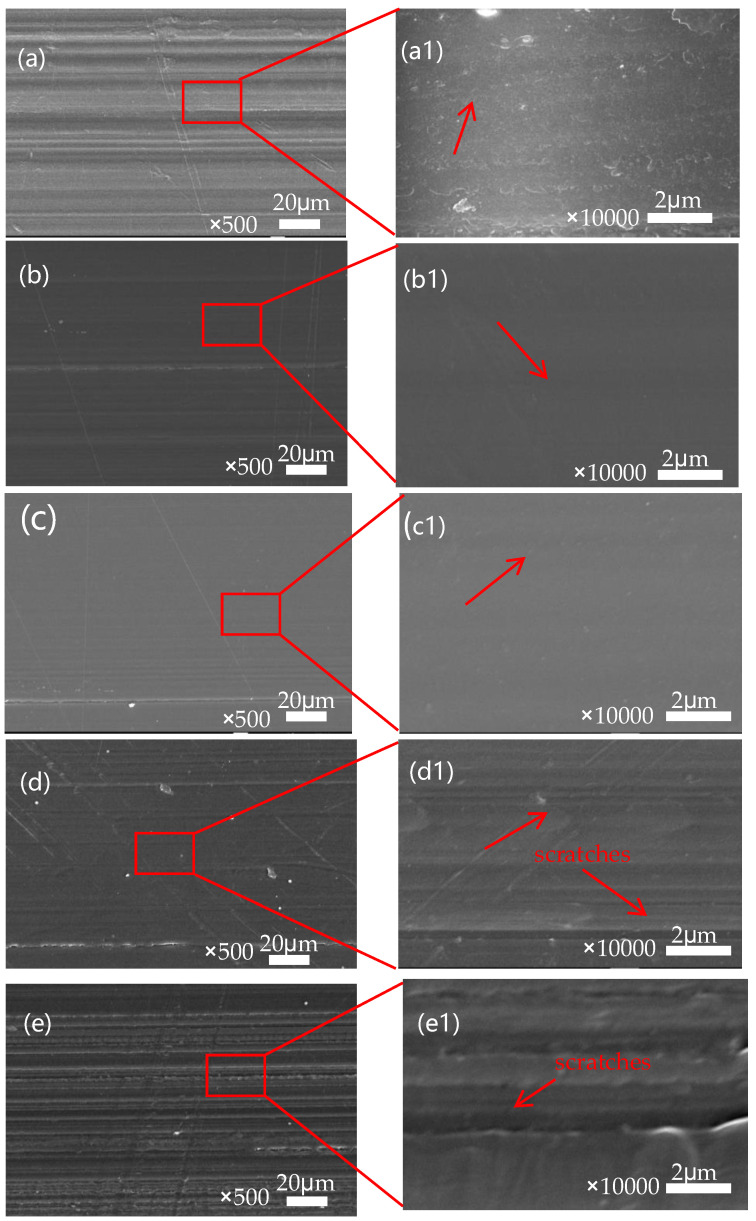
SEM images of the worn surfaces of the SPZ samples with different sulfonation times in contact with GCr15 after friction. These images include SPZ-2 min (**a**), SPZ-3 min (**b**), SPZ-4 min (**c**), SPZ-5 min (**d**), and SPZ-8 min (**e**). Additionally, the corresponding enlarged views are shown in (**a1**–**e1**).

The worn surface morphology of SPZ under dry friction conditions are shown in Figure 9, and enlarged images are also provided. From the observation of the worn surfaces, it can be concluded that as the sulfonic acid group density increased, the density of the grooves on the material surface also increased. In the localized enlarged images of the samples (a1–e1) in Figure 9, we can observe some tiny nanogrooves on the SPZ surface, which were parallel to the sliding direction of the material. During the friction process, some hard nanoparticles were embedded in the transfer film layer on the dual surface, resulting in some scratches. The width of these scratches is approximately in the range of 300–400 nanometers, while the diameter of the nanoparticles is around 500 nanometers. In this case, the width of the scratches was almost identical to the size of the nanoparticles. For these hard particles, sliding and rolling typically occurred simultaneously, meaning that nanoparticles mainly helped reduce friction through a rolling effect, thereby enhancing the wear resistance of the composite material, a view supported by previous research [22].

**Figure 10:**
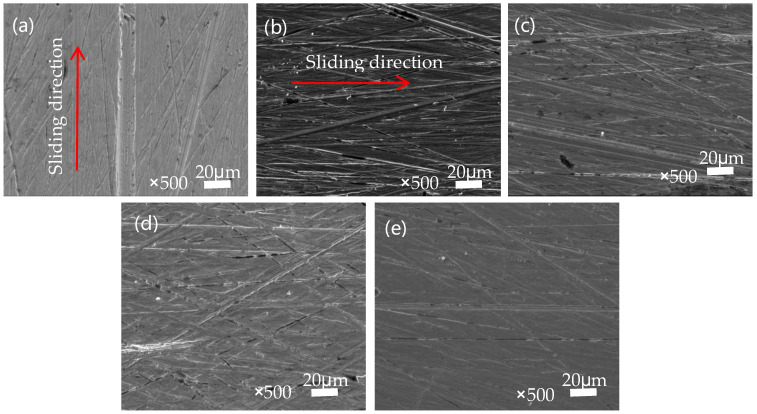
The microscopic morphology of the transfer film after mutual abrasion between SPZ and GCr15. (**a**) SPZ-2 min; (**b**) SPZ-3 min; (**c**) SPZ-4 min; (**d**) SPZ-5 min; (**e**) SPZ-8 min.

According to the display in Figure 10, we can observe the counterpart wear of SPZ under dry friction with different sulfonation times. From the observation in Figure 10a, distinct furrows are evident after 2 min of sulfonation, primarily due to abrasive wear. Furthermore, the thin layer formed by the transfer film appears thin and uneven. With prolonged sulfonation time, as observed in Figure 10b–e, the scratches on the counterpart surface become increasingly uniform, accompanied by more subtle scratches, mainly attributed to mild abrasive wear. It is speculated that this leads to more particles transferring between the contacting mating surfaces. The ZIF-8 nanoparticles released during frictional processes act on the transfer film, promoting the transformation of carbon elements in the transfer film towards an ordered direction. These particles easily undergo chelation reactions with the metal counterparts, enhancing the bonding between the transfer film and the metal mating surfaces. The presence of chemical bonds between the transfer film and the metal mating surfaces promotes the formation of the transfer film. This process helps to fill the surface irregularities of the counterpart surfaces, gradually increasing the thickness of the transfer film. As a result, the transfer film on the mating surface becomes more uniform, of higher quality, and more complete, thereby enhancing its load-bearing capacity. These improvements significantly enhance the wear resistance of the sample.

#### 3.3.3. The Influence of Different Loads and Velocities on Tribological Performance

**Figure 11:**
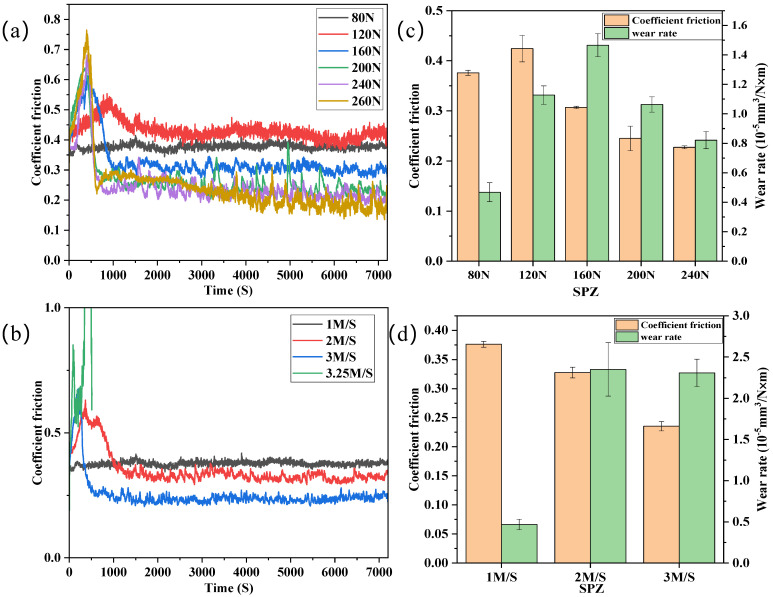
The friction coefficient curves over time (**a**,**b**) and steady−state friction coefficient plots with characteristic wear rates (**c**,**d**) for SPZ under various loads and velocities.

Figure 11 presents the average friction coefficients and wear rates of SPZ (with a filling density of 90% and a sulfonation time of 8 min) under different loads (with a fixed velocity of 1 m/s) and different velocities (with a fixed load of 80 N) after wear testing. The results indicated that the friction coefficients for the samples in Figure 11a exhibited an initial increase followed by a decrease under varying loads. However, the friction curve for the 260 N load showed significant fluctuations after 60 min, attributed to changes in the counterpart and severe frictional oxidation of the counterpart surface. In Figure 11b, it can be observed that the friction coefficients gradually decreased with increasing velocity. When the velocity reached 3.25 m/s, there was a rapid increase in the friction coefficient within a short time, which can be attributed to surface softening due to elevated temperatures, leading to frictional invalidation [23].

From Figure 11c,d, it can be observed that the wear rate of SPZ is lowest at low loads and low speeds. Under different load conditions, the friction coefficient shows a trend of initially increasing and then decreasing with the increase in load, reaching its maximum at 120 N, followed by a decrease. The decrease is attributed to the increase in friction surface temperature at high loads, leading to the transition of the composite material from high elasticity to viscous flow state, thereby reducing the friction coefficient. The wear rate initially increases with the increase in load, reaching its maximum at 160 N, and then decreases. This is because at high loads, wear debris is compacted onto the corresponding surface, resulting in a decrease in wear rate. With the increase in speed, the wear rate remains relatively unchanged at 2 m/s and 3 m/s, compared to 1 m/s, but significantly increases at higher speeds. It is speculated that solid nanoparticles are easily transferred to their metal counterpart under low-speed conditions, while lubrication failure of the transfer film is more likely to occur at high speeds [24].

**Figure 12:**
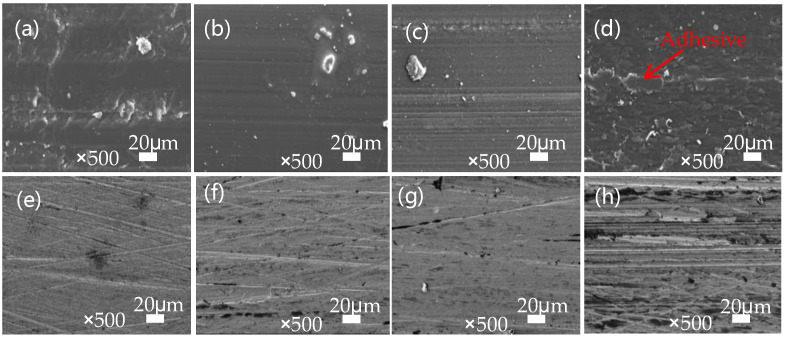
The SEM images of the worn surfaces of SPZ (with a filling density of 90% and a sulfonation time of 8 min) under different loads (120 N (**a**), 160 N (**b**), 200 N (**c**), 240 N (**d**)), as well as the microscopic morphology of the transfer films after wear testing under 120 N (**e**), 160 N (**f**), 200 N (**g**), and 240 N (**h**) loads.

Figure 12a–d present the morphological analysis of the polymer wear tracks on SPZ under different load conditions after sliding against GCr15. It can be observed that at 120 N, the wear track surface was mainly characterized by adhesive wear resulting from adhesion. At 160 N and 200 N, the surface appeared relatively smooth with some frictional debris particles, accompanied by minor furrows along the direction of sliding. At 200 N, this may be attributed to the increased load, causing ZIF-8 particles to be compacted onto the sample surface and within gaps, primarily existing in a sliding manner rather than rolling, resulting in more furrows on the surface. Under the condition of 240 N, wear debris was compacted onto the worn surface due to the elevated contact temperature. This is accompanied by increased adhesive behavior between the sample and the counterpart, leading to the formation of sheet-like debris adhered to the worn surface [25].

As shown in Figure 12e–h, the SEM images depict the transfer films formed on the counterpart surfaces after SPZ and GCr15 pairings under different loads. Under low-load conditions, the transfer film was relatively thin. From Figure 12h, it can also be observed that adhesion phenomena became more pronounced as the sample underwent heavy-load sliding. In the case of 240 N, the transfer film was thick but uneven due to adhesive behavior, and the formed transfer film effectively contributed to reducing the wear rate of the material.

**Figure 13:**
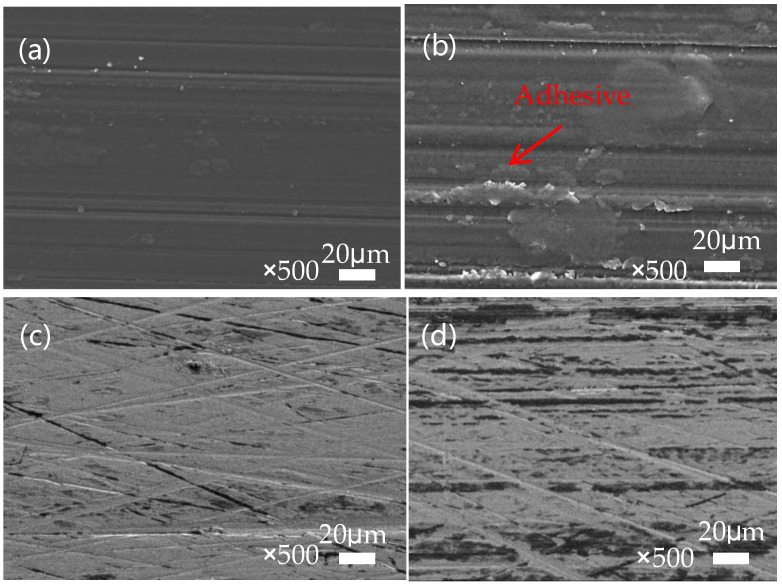
SEM images of the worn surfaces after friction between SPZ and GCr15 at different velocities, 2 m/s (**a**) and 3 m/s (**b**). Additionally, it shows SEM images of the microstructure of the transfer films formed on the counterpart surfaces after frictional contact at velocities 2 m/s (**c**) and 3 m/s (**d**).

In order to understand the variation in wear mechanisms with changing velocity, scanning electron microscopy (SEM) was employed to characterize the microphotographs of the worn surfaces and counterparts, which are shown in Figure 13. As can be seen from Figure 13a,b, with the increased velocity, the surface of the sample experienced increasingly severe scratching along the direction of sliding, leading to formation of large fragments. In Figure 13c,d, it can be observed that the formed transfer film was thin and uneven, with evident scratching. This may be attributed to the fact that as the velocity increased, nanoparticles were unable to transfer to the counterpart surface in a timely manner. Consequently, the scratches caused by the nanoparticles on the counterpart surface became more pronounced, resulting in the absence of a high-quality and uniform transfer film, which induced more significant wear under high-speed conditions.

### 3.4. Analysis of the Chemical Structure of Transfer Membranes

**Figure 14:**
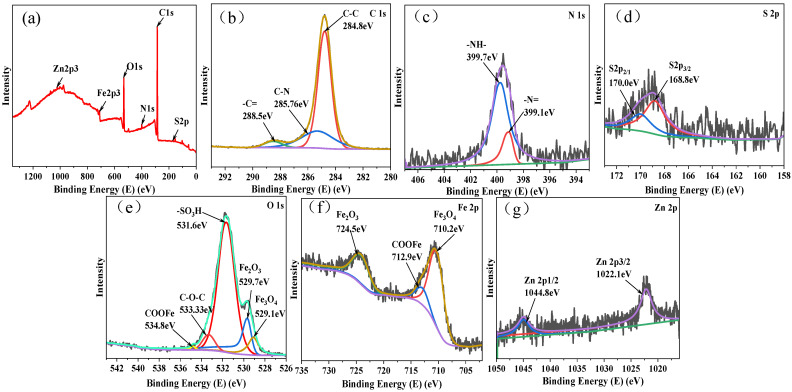
The X-ray photoelectron spectroscopy (XPS) spectra of the transfer film formed after the wear test between SPZ and GCr15. The spectra includes the overall survey (**a**), C1s (**b**), N1s (**c**), S1s (**d**), O1s (**e**), Fe2p (**f**), and Zn2p (**g**) regions.

Figure 14 presents the X-ray photoelectron spectroscopy (XPS) spectra of the transfer films formed after dry sliding. Initially, in the full XPS spectrum (Figure 14a), peaks corresponding to C1s and O1s were observed on all substrate surfaces. After sulfonation, additional S, N, and Zn peaks were detected, indicating the successful incorporation of −SO_3_H and Zn^2+^ ions on the PEEK matrix [26]. The spectra exhibited peaks at 284.8 eV, 288.5 eV, and 285.76 eV, corresponding to C in the aromatic rings and C=O groups in PEEK molecules [27], and C-N bonds in the imidazole rings of 2-methylimidazole [28]. Figure 14c displays the N1s spectrum with peaks at 399.1 eV and 399.7 eV assigned to −N= and −NH−, respectively, which are attributed to ZIF-8 and the pyridine nitrogen of the imidazole ligand [29]. The S2p3/2 and S2p1/2 binding energy peaks at 168.8 eV and 170.0 eV in Figure 14d corresponded to the −SO_3_H groups, confirming the successful sulfonation of the PEEK matrix [26]. The Fe2p spectrum exhibits binding energies at 710.2 eV and 724.5 eV, and the O1s spectrum shows peaks at 529.1 eV and 529.7 eV (Figure 14f), indicating the occurrence of metal–tribological oxidation reactions. Additionally, the peaks at 712.9 eV in the Fe2p spectrum and 534.8 eV in the O1s spectrum signify the formation of metal–organic bonds, specifically chelation reactions between peroxides generated in the PEEK chain segments friction and the metallic counterpart [30,31]. Furthermore, the O1s spectrum exhibits peaks at 531.6 eV and 533.33 eV, corresponding to the sulfonate groups formed after sulfonation and the ether bonds in PEEK [32]. Figure 14g displays the Zn2p spectrum with two fitted peaks at 1022.1 eV and 1044.8 eV, corresponding to Zn2p3/2 and Zn2p1/2 [33].

In conclusion, the C1s spectrum at 285.76 eV represented the C-N bonds in the 2-methylimidazole rings, and the peaks at 531.6 eV and 533.33 eV in the Fe2p and O1s spectra, respectively, corresponding to the sulfonate groups and ether bonds in PEEK. The Zn2p spectrum with peaks at 1022.1 eV and 1044.8 eV clearly indicates the successful transfer of ZIF-8 nanoparticles onto the counterpart during the friction process. Therefore, the ZIF-8 nanoparticles in the transfer film play a predominant role in reducing friction.

**Figure 15:**
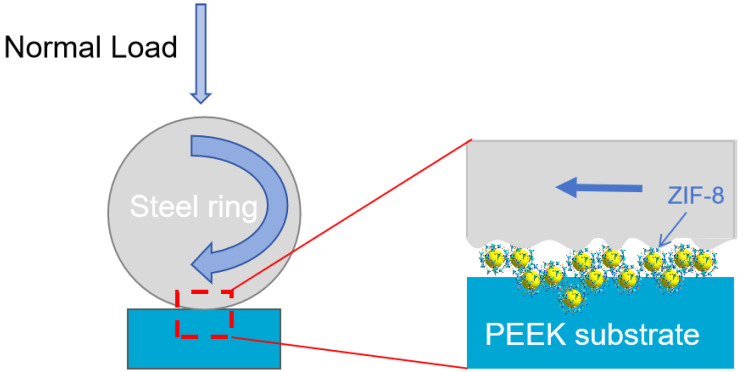
Wear Mechanism of the SPZ Material.

Based on the above results, with the friction experiment, nanoscale ZIF-8 particles can easily enter the sliding contact region between the sample and counter steel surface. Within this contact area, ZIF-8 particles played a rolling role in both the smooth and raised regions (as shown in Figure 15), effectively acting as a third-party medium, which efficiently prevents adhesive wear between the contacting surfaces [34,35]. Simultaneously, ZIF-8 fills the recessed areas of the surface, making the friction surface smoother, further reducing the likelihood of wear. As sliding progresses, ZIF-8, along with iron oxide, forms a friction film covering the friction interface, thereby enhancing the overall friction performance [36].

## 4. Conclusions

Through systematic studies, we confirmed the successful formation of ZIF-8 nanoparticles on the surface of PEEK using a simple and efficient in situ synthesis method. The comprehensive investigation of the effects of different filling densities, sulfonation times, loads, and speeds on the frictional performance of PEEK and SPZ led to the following conclusions:

1. Under dry friction conditions, it was observed that different filling densities exhibited a synergistic effect between filling density and nanoparticles. The frictional performance of SPZ with a filling density of 90% was significantly better than that of other filling densities. At a filling density of 90%, the wear rate of SPZ decreased by 40%. This improvement was attributed to some nanoparticles being filled into the gaps on the sample surface during friction, enhancing its load-bearing capacity and promoting the transfer of wear debris to the mating surface, thereby improving the tribological performance of the composite material.
2. Increasing sulfonation time significantly reduced the wear rate of the composite material. Particularly, at a sulfonation time of 8 min, the wear rate of SPZ decreased by 120% compared to PEEK, demonstrating the best anti-wear effect. The results indicated that with the increase in sulfonation time, the grafting sites on the PEEK matrix increased, resulting in excellent tribological performance of the composite material.
3. Analysis and examination of SPZ under different loads and speeds revealed that SPZ exhibited the best tribological performance under low load and low-speed conditions. This was attributed to the easier release of surface nanoparticles into the friction interface under low-load and low-speed conditions, exerting excellent anti-friction and wear effects. Under high-load and high-speed conditions, nanoparticles were compacted onto the sample surface and into the gaps, where ZIF-8 nanoparticles did not roll effectively. Instead, they caused noticeable scraping of the sample and transfer film, resulting in deteriorated tribological performance.

## Figures and Tables

**Table 1:** 3D printing parameters for PEEK.

Parameter	Value
Nozzle temperature	440 °C
Bed temperature	120 °C
Cavity temperature	90 °C
Nozzle diameter	0.4 mm
Layer thickness	0.2 mm
Printing speed	30 mm/s
Filling angle	45°/−45°
Filling density	70%, 75%, 80%, 85%, 90%
Cooling method	Air pipe

## Data Availability

The raw data supporting the conclusions of this article will be made available by the authors on request.
